# Prebiotic Phosphorylation of Uridine using Diamidophosphate in Aerosols

**DOI:** 10.1038/s41598-019-49947-8

**Published:** 2019-09-19

**Authors:** A. D. Castañeda, Z. Li, T. Joo, K. Benham, B. T. Burcar, R. Krishnamurthy, C. L. Liotta, N. L. Ng, T. M. Orlando

**Affiliations:** 1 School of Chemistry and Biochemistry, Georgia Institute of Technology, Atlanta, GA 30332 USA; 2 School of Earth and Atmospheric Sciences, Georgia Institute of Technology, Atlanta, GA 30332 USA; 3 Department of Chemistry, The Scripps Research Institute, La Jolla, CA 92037 USA; 4 School of Chemical and Biomolecular Engineering, Georgia Institute of Technology, Atlanta, GA 30332 USA

**Keywords:** Origin of life, Mass spectrometry, Origin of life, Atmospheric chemistry

## Abstract

One of the most challenging fundamental problems in establishing prebiotically plausible routes for phosphorylation reactions using phosphate is that they are thermodynamically unfavorable in aqueous conditions. Diamidophosphate (DAP), a potentially prebiotically relevant compound, was shown to phosphorylate nucleosides in aqueous medium, albeit at a very slow rate (days/weeks). Here, we demonstrate that performing these reactions within an aerosol environment, a suitable model for the early Earth ocean-air interface, yields higher reaction rates when compared to bulk solution, thus overcoming these rate limitations. As a proof-of-concept, we demonstrate the effective conversion (~6.5–10%) of uridine to uridine-2′,3′-cyclophosphate in less than 1 h. These results suggest that aerosol environments are a possible scenario in which prebiotic phosphorylation could have occurred despite unfavorable rates in bulk solution.

## Introduction

The phosphorylation of prebiotic molecules is essential for the emergence of life. These reactions, especially those pertaining to the phosphorylation of nucleosides, have been widely investigated in the context of origins of life research^1–9^. One of the fundamental problems in establishing prebiotically plausible phosphorylation routes is that water, the most ubiquitous solvent on Earth, provides an environment that renders phosphorylation reactions thermodynamically unfavorable^7^. Thus, many of these studies employ the use of condensing agents^1,3^ or alternative environments^5,7,8^.

Recently, Krishnamurthy and co-workers^10^ demonstrated phosphorylation of various prebiological molecules (nucleosides, amino acids, and lipid precursors) using diamidophosphate (DAP) in aqueous conditions. DAP has been previously employed as a prebiotic phosphorylation reagent^11–13^, and it can be synthesized via corrosion of schreibersite with aqueous ammonia^14^ or reacting prebiotically available trimetaphosphate^15^ with ammonia^16,17^. In this study they demonstrated effective, one-pot phosphorylation reactions in both bulk solutions, and so-called “paste” conditions where they added only ~20–30 µL of water. Through these conditions, they demonstrated phosphorylation of nucleosides, amino acids, and lipid precursors. The “paste” conditions gave the benefit of using water as a solvent while maintaining low water activity to lower the rate of hydrolysis. They found that the highest yields were observed in these paste reactions. Uridine, for example, was converted to ~89% uridine-2′,3′-cyclophosphate (2′,3′-cUMP) in 18 days, while comparable reactions in bulk solution yielded only ~29% conversion in 30 days. They established that their yields were dependent not only on the volume of water (solutions vs. paste), but also on the pH of solutions, concentration of divalent cations added, and the presence of imidazole, which not only enabled the phosphorylation reaction but also seemed to suppress hydrolysis/self-condensation of DAP. Imidazole has been shown to activate DAP by forming the imidazolide derivative^10^, and its formation has been demonstrated as prebiotically plausible^18,19^.

In this study, we extend DAP-based phosphorylation to an aerosol environment. Atmospheric aerosols would have offered many advantages as chemical reactors on the prebiotic Earth^20,21^. Aqueous/organic droplets forming at the water-air interface, (i. e. ocean-air for example), would have enabled high concentrations of potential organic reactants that are unattainable in the bulk ocean. Microdroplets have also been shown to accelerate biochemical reactions, with rates that are observed to increase up to 10^6^-fold when compared to bulk solutions^22^. Various studies have suggested that the vesicle or water-air interfaces provide favorable environments for prebiotic synthesis^9,21,23,24^. Notably, Zare and co-workers recently detected the formation of sugar-phosphate and uridine ribonucleoside in aqueous microdroplets using phosphoric acid^9^. They used an electrospray ionization (ESI) source for form the droplets, and analyzed the products via mass spectrometry. In the absence of an applied voltage, phosphorylated products were still detected, indicating that the thermodynamically unfavorable reaction had occurred in the aerosol and not as a consequence of applied energy.

In contrast, our work produces and directly injects aerosols that contain DAP, a prebiotically plausible phosphorylating agent^12,13^, into an enclosed Teflon reaction chamber. Following a system similar to that established by Krishnamurthy and co-workers^10^, a solution mixture of uridine and DAP is atomized into poly-dispersed aerosols with a mode of ~100 nm. We hypothesized that an aerosol environment would mimic “paste” conditions, as both environments feature rapid water evaporation and increased concentrations of the reagents, and may result in a faster rate of reaction compared to previous results. We report here that aerosols do have a positive effect on the phosphorylation reaction rates and observed significant formation of 2′,3′-cUMP in the aerosol-mediated reactions.

## Results

### Concept and aerosol formation

**Figure 1:**
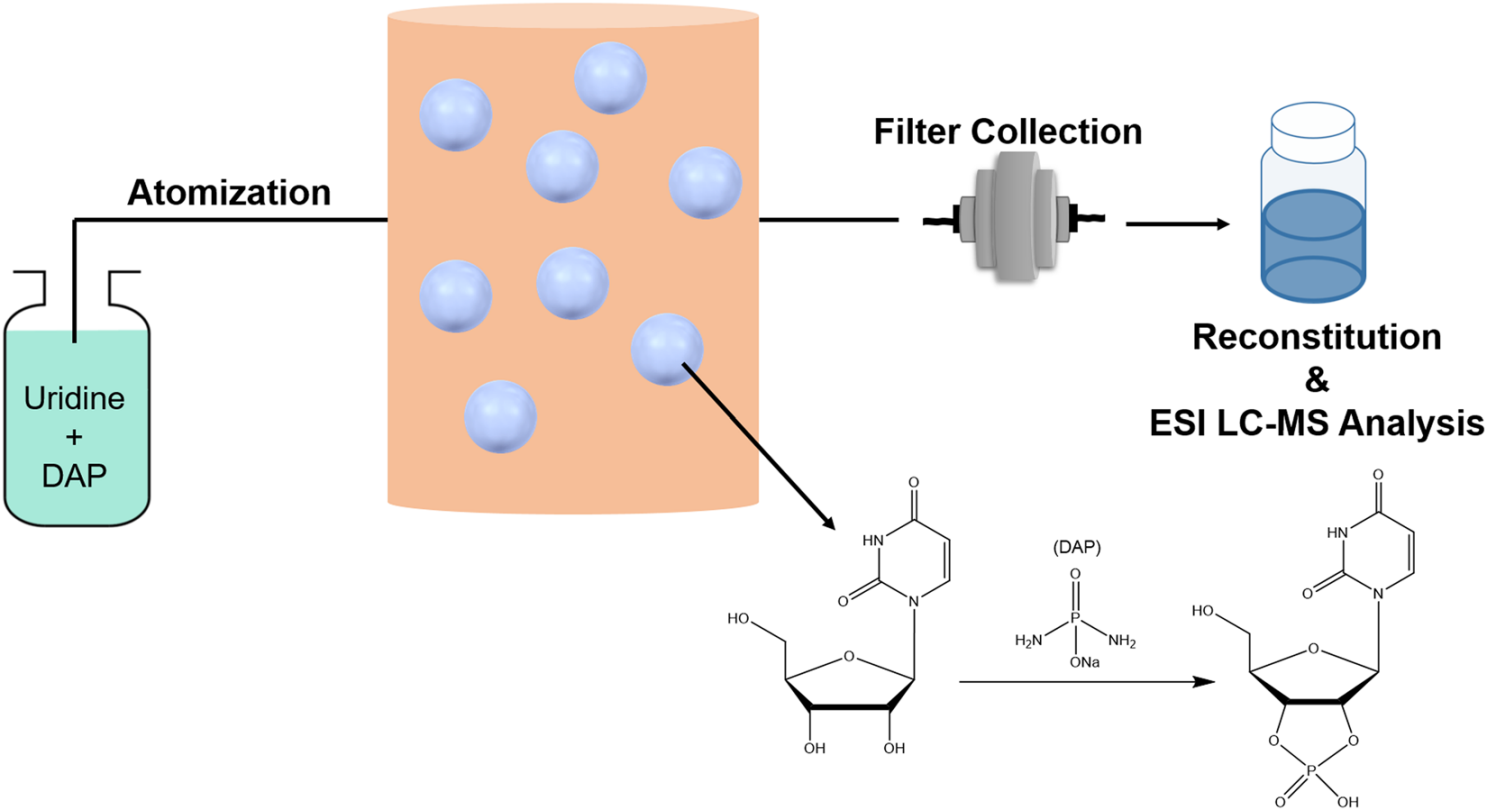
A solution of uridine and DAP is atomized into a Teflon chamber, where the aerosol particles are suspended until collection. Following collection on a filter, products are reconstituted in H_2_O and injected into an ESI LC-MS for analysis.

The overall concept of our experiments is shown in Fig. 1. Uridine and DAP were combined in solutions adjusted to pH 5.5 using HCl, followed by 20 min atomization of these solutions to form the aerosol phase, where the particles were suspended in an enclosed Teflon chamber. 2′,3′-cUMP was formed within the aerosols that were collected on a Teflon filter, reconstituted with H_2_O, and analyzed using liquid chromatography mass spectrometry (LC-MS).

**Figure 2:**
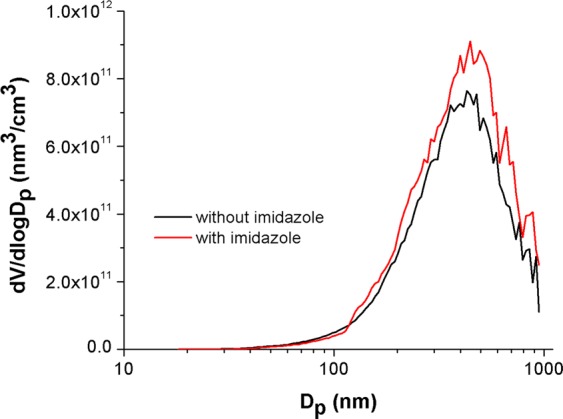
Particle volume distribution inside the Teflon chamber (sampled after 20 min of aerosol generation) from atomized solutions without (black) and with (red) 1 eq imidazole.

Total particle number concentrations of ~1 × 10^5^ particles/cm^3^ were achieved within 20 min atomization. The resulting aerosol mass concentration was sufficient for product collection onto Teflon filters and subsequent off-line analysis. Figure 2 shows the particle volume distributions at 20 min for aerosols atomized from a solution without (black line) and with (red line) 1 eq imidazole added. The mode was ~90 nm for experiments without imidazole and ~120 nm for those with imidazole present, respectively. The addition of imidazole increases the volume and size of the particles while the effective and accessible surface to volume ratio decreases. Since the rates are higher for larger droplets, this is likely related to the increased density of imidazole and encounter frequency at the surface which can result from the Kelvin Effect^25^. The Kelvin equation describes the change in vapor pressure of a curved droplet when compared to a flat surface. It defines the vapor pressure as inversely proportional to the radius of the droplet. Here, the larger droplets containing imidazole yield more molecules immediately adjacent to a molecule on the surface compared to the smaller droplets without imidazole.

### Collected aerosol analysis

**Figure 3:**
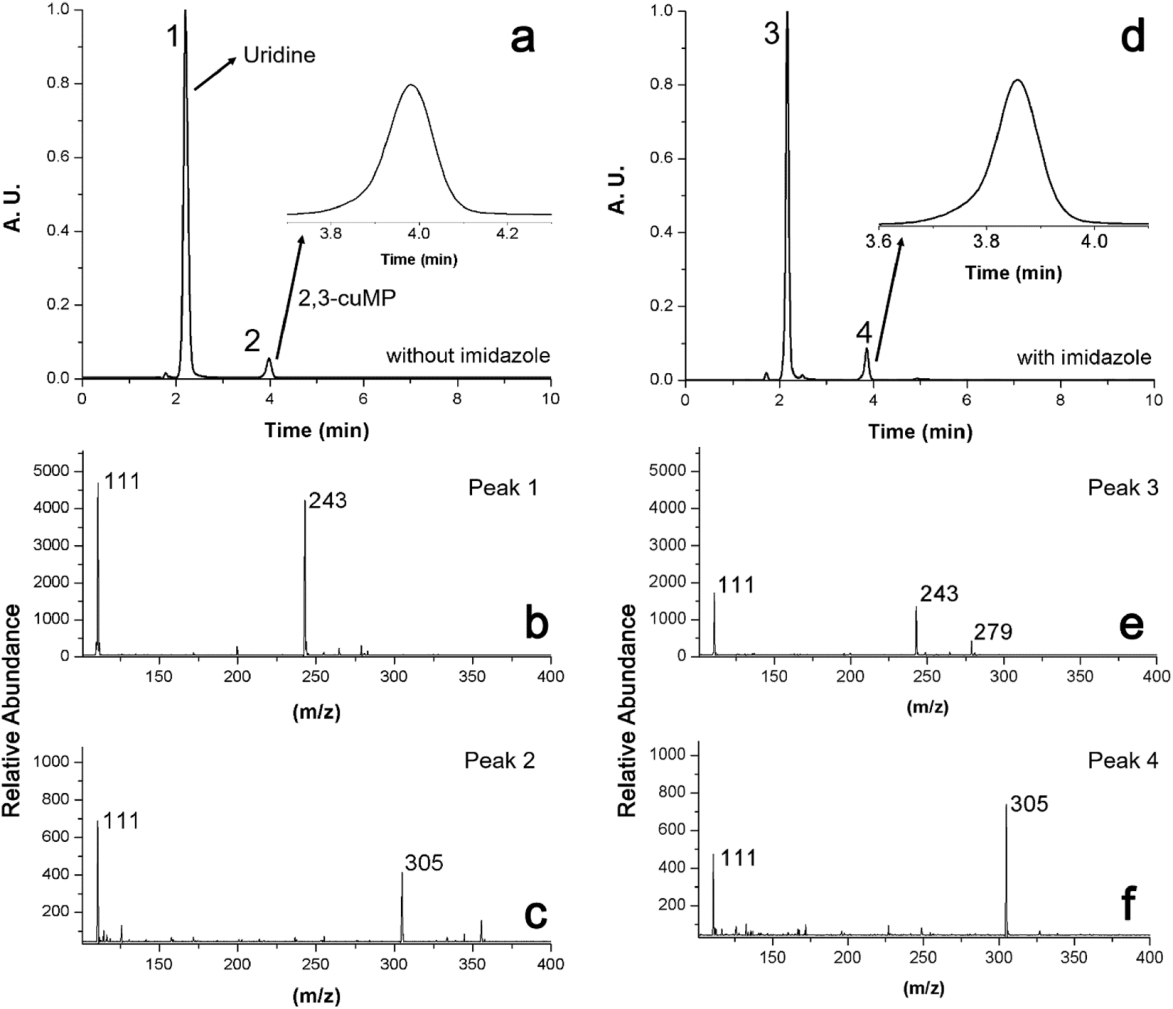
LC-MS data of collected and reconstituted aerosols. (**a**) Liquid chromatogram showing the presence of uridine (peak 1) and product 2′,3′-cUMP (peak 2) in aerosols formed without imidazole. The identity of the compounds is verified via negative-mode ESI-MS in (**b**) and (**c**) for peak 1 and peak 2, respectively. (**d**) Liquid chromatogram showing the presence of uridine (peak 3) and product 2′,3′-cUMP (peak 4) in aerosols formed with the inclusion of 1 eq imidazole. The identity of the compounds is verified via negative-mode ESI-MS in (**e**) and (**f**) for peak 3 and peak 4, respectively.

Aerosols from solutions of 1 eq uridine + 5 eq DAP + 3 eq MgCl_2_, pH adjusted to 5.5 with and without 1 eq of imidazole were collected on Teflon filters, reconstituted in 100 µL H_2_O, and analyzed using ESI LC-MS. These results, which were obtained after suspending the aerosols in the Teflon chamber for 50 minutes, are shown in Fig. 3. Figure 3a shows the chromatography of the collected aerosols without the presence of imidazole. Uridine eluted at ~2.3 min, followed by 2′,3′-cUMP at ~4 min. No other species were detected in the experimental runs. For comparison, in addition to the 2′,3′-cUMP, Krishnamurthy and co-workers detected small amounts of the 5′-amidophosphate in their paste conditions^10^. In their work, monitoring by ^31^P-NMR (nuclear magnetic resonance) showed that the major species observed could be attributed to phosphorylation on the 2′- and 3′-oxygen atoms of the sugar moiety and not on the 5′-OH nor on the -NH moieties of the uracil nucleobase. In our study, sufficient mass could not be obtained for tracking via ^31^P-NMR. Thus, we have relied on LC-MS analysis, which did not indicate the presence of other phosphorylated products. The retention times of both compounds (uridine and 2′,3′-cUMP) were found to deviate slightly from run to run. This is most likely due to the nature of our ion pairing method (see LC-MS Methods). The percent conversion of uridine to 2′,3′-cUMP was 6.5 ± 1.3% under these conditions based on the percent area under the product peak relative to the total peak areas. The identity of the compounds was confirmed using ESI-MS, as shown in Fig. 3b,c. Figure 3b shows the expected molecular ion peak at m/z 243 corresponding to deprotonated uridine, followed by a loss of ribose leaving uracil at m/z 111. Figure 3c shows the product peak for 2′,3′-cUMP at m/z 305, followed by a similar loss of ribose to yield uracil at m/z 111. The chromatogram for collected aerosols of solutions including imidazole are shown in Fig. 3d, and the corresponding mass spectrometry for the peaks is shown in Fig. 3e,f. Once again, the molecular ion peak for uridine is observed at m/z 243, followed by a loss of ribose yielding uracil at m/z 111 (Fig. 3e). A chloride adduct of uridine is also detected at m/z 279. Figure 3f confirms the presence of the product peak for 2′,3′-cUMP at m/z 305 and uracil at m/z 111 following the loss of ribose. In these experiments, the percent conversion of uridine to 2′,3′-cUMP was 10.2 ± 0.4%.

Chromatograms were also obtained by analyzing stock solutions with and without uridine (see Supplementary Fig. S2). In analyzing the stock solutions, whether imidazole is added or not, only uridine is eluted and no product peaks were observed.

### Control experiments

**Figure 4:**
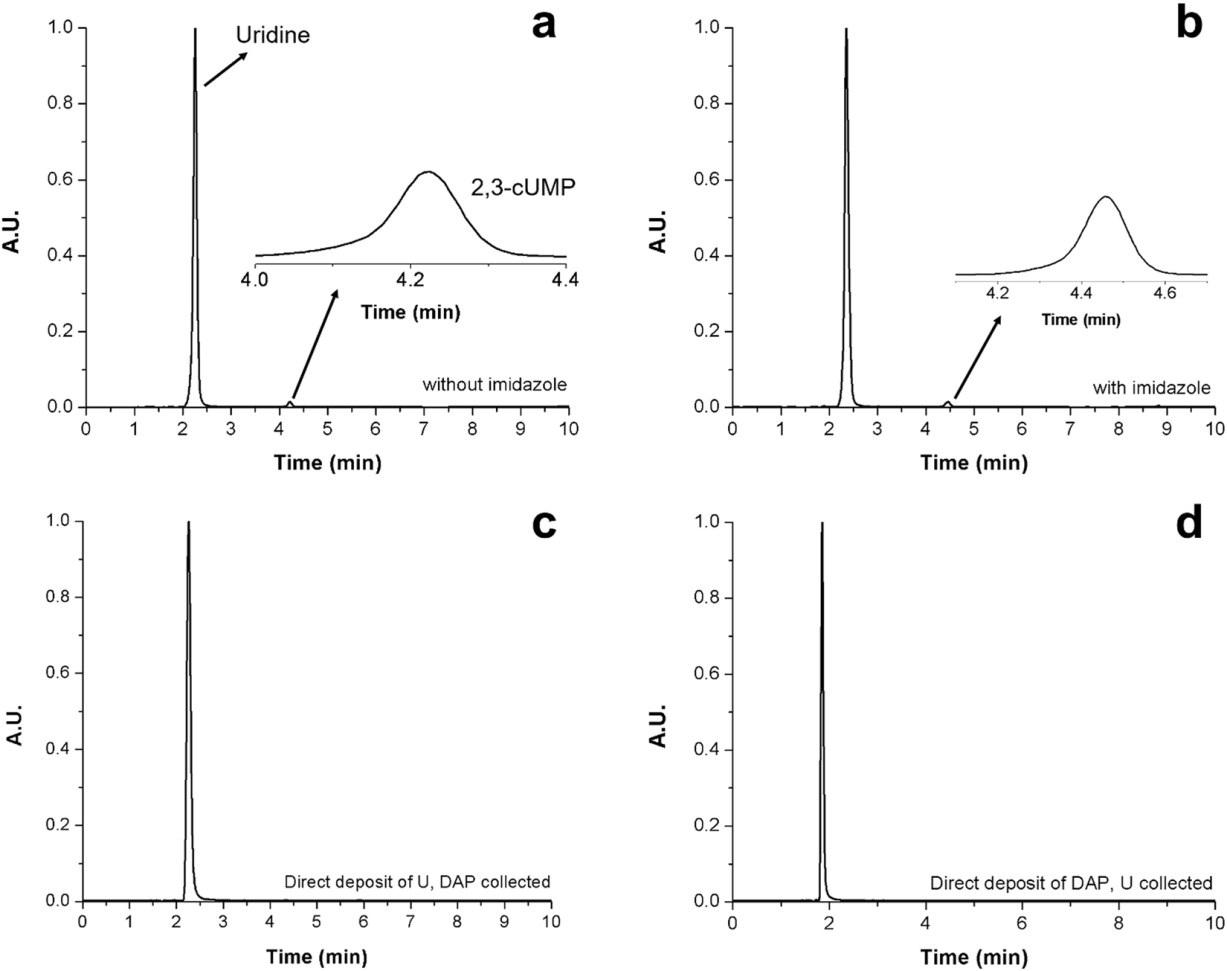
LC chromatograms from control experiments to determine contribution from solution dry-down on Teflon filters. Collected aerosols have been reconstituted in 100 µL H_2_O. (**a**) 5 min atomization of a solution of 1 eq uridine + 5 eq DAP + 3 eq MgCl_2_, pH adjusted to 5.5 deposited directly onto the filter surface. The 2′,3′-cUMP product percent from these runs was 1.7 ± 0.3%. (**b**) 5 min atomization of a solution of 1 eq uridine + 5 eq DAP + 3 eq MgCl_2_ + 1 eq imidazole, pH adjusted to 5.5 deposited directly onto the filter surface. The 2′,3′-cUMP product percent from these runs was 1.9 ± 0.1%. (**c**) 5 min atomization of a solution of 1 eq uridine + 3 eq MgCl_2_, pH 5.5 deposited directly onto the filter surface, followed by collection of an aerosol of 5 eq DAP and 1 eq imidazole from the bulk chamber. No product was detected. (**d**) 5 min atomization of a solution of 5 eq DAP and 1 eq imidazole deposited directly onto the filter surface, followed by collection of an aerosol of 1 eq uridine + 3 eq MgCl_2_, pH 5.5 from the bulk chamber. No product was detected.

Although formation of 2′,3′-cUMP was observed in the collected aerosols, we were aware that these experiments do not conclusively prove that the reaction occurred in the aerosol phase. To answer the question of whether the process of depositing the aerosols on the Teflon filter had any contribution to driving the reaction, we performed control experiments. Depositing a solution of 1 eq uridine + 5 eq DAP + 3 eq MgCl_2_, pH adjusted to 5.5 without and with 1 eq imidazole (see Supplementary Fig. S3) for 50 min (approximately the length of one aerosol experiment from atomization to LC-MS injection) directly on Teflon filters did not yield any product peak in the chromatograms. Only the peak corresponding to uridine was observed. We then investigated whether the aerosol “residence time”, or the time that the aerosol is suspended in the chamber (~50 min total) had any effect on product formation. Figure 4a,b show experiments in which solutions without (4a) and with 1 eq imidazole (4b) were atomized and deposited directly onto the filter surface, rather than suspending the aerosols in the Teflon chamber. The total atomization time was 5 min. In both cases, a minimal amount of product was formed: 1.7 ± 0.3% in solutions without imidazole, and 1.9 ± 0.1% in solutions with imidazole. These suggested that in typical experiments, reactions were indeed taking place in the aerosol phase to form the corresponding products.

To verify that all components must be present in the aerosol phase to observe products, a series of experiments were conducted in which one of the two major components (uridine or DAP) was deposited directly onto the filter surface, followed by collection of aerosols consisting of the other component from the chamber. Any product formed under these conditions must be formed on the filter surface. These results are shown in Fig. 4c,d. Figure 4c shows the chromatogram obtained from direct atomization of a solution 1 eq uridine + 3 eq MgCl_2_, pH 5.5 onto the filter, followed by collection of aerosols of 5 eq DAP and 1 eq. imidazole from the Teflon chamber. No product peak corresponding to 2′,3′-cUMP was observed. Figure 4d shows the chromatogram obtained from direct atomization of a solution 5 eq DAP and 1 eq imidazole onto the filter, followed by collection of aerosols of 1 eq uridine + 3 eq MgCl_2_, pH 5.5 from the Teflon chamber. Once again, no product peak corresponding to 2′,3′-cUMP was observed.

Lastly, it was necessary to investigate whether the voltage provided by the electrospray (3 kV) nozzle could provide energy to drive the reaction after the collection and reconstitution process. Thus, we performed direct inject negative mode ESI-MS on the stock solutions. The resulting mass spectra of solutions without and with imidazole (see Supplementary Fig. S4) show no peak at m/z 305 corresponding to 2′,3′-cUMP. This confirms that the energy from the ionization process is not responsible for the observed phosphorylation reactions.

## Discussion

The water-air interface is a valuable model to consider when studying prebiotic phosphorylation. More than 90% of the early Earth was covered in water, making the presence of aqueous atmospheric aerosols even more prevalent than they are today^23^. We have shown rapid reaction rates of uridine using DAP in a model aerosol environment when compared to bulk. There are many factors that are thought to contribute to the accelerated reaction rates achieved in microdroplets versus a bulk environment^26^. One explanation is that there is increased solvent evaporation rates, which contributes to higher concentrations of reagents in proximity to each other due to the Kelvin Effect^25^. Because of the high evaporation rate of water, the water activity within the aerosol and on the aerosol-air interface is greatly decreased when compared to bulk conditions. However, there are solvation waters left for reactants within the aerosol, and the reactions are likely governed by both the water activity (related to pH) and the presence of imidazole.

Another, possibly more significant contribution, is that an aerosol droplet provides a high surface-to-volume ratio, and is thus a unique polar surface environment. Similarly, the effect of pH should be considered. It is well known that the pH of a given aerosol differs from that of its bulk origin^27,28^. Many prior aerosol studies have observed increased organic aerosol formation and higher reaction rates for condensation and heterogeneous reactions in acidic aerosols^25,29^ This is consistent with our results obtained with a solution initially adjusted to pH 5.5 using HCl.

The residence time of aerosol droplets in the chamber appears to be a contributing factor to achieving increased phosphorylation rates in this study. This is demonstrated by our results obtained after 5 min atomization and direct deposition of aerosols onto the filter, which yielded only ~1% 2′,3′-cUMP regardless of imidazole addition or not. In contrast, aerosols that were suspended in the chamber for 50 min yielded ~6.5 and ~10.2% 2′,3′-cUMP without and with imidazole, respectively. For comparison, bulk solution experiments at pH 5.5 reported by Krishnamurthy and co-workers yielded ~7% conversion of uridine in 9 days^10^. The results reported here also suggest that longer time-based studies would produce higher yields of 2′,3′-cUMP. Such studies proved to be a challenge with our current setup and chamber volume. Over the course of the chamber experiments, particles are continuously lost to chamber walls, which is typical in any aerosol chamber experiments^30^. Though we are limited by the dimensions of our aerosol reaction chamber, wall losses are minimized in larger chambers with a larger surface-area-to-volume ratio and longer residence times can be achieved.

The inclusion of imidazole in the reaction conditions was found to have a significant effect on the percent formation of 2′,3′-cUMP, with aerosols that contained imidazole producing higher yields of product. Imidazole was previously found to decelerate the hydrolysis/condensation of DAP observed in solutions of pH 7 or lower through formation of an amidophosphoroimidazolide intermediate^10^. These results are consistent with the findings presented in this work, as collected aerosols with imidazole added produced ~4% more 2′,3′-cUMP compared to aerosols without imidazole. The addition of imidazole provides an environment in which DAP is more readily available for phosphorylation, as it slows down the hydrolysis of DAP, activates DAP to form the imidazolide derivative, and thus enhances phosphorylation yields.

Through the series of control experiments, we have demonstrated that the phosphorylation reaction occurs in the aerosol phase and not as a consequence of deposition on the filter surface. Furthermore, we have shown that voltage applied during the ESI process does not contribute to product formation. Ideally, future studies of these reactions would implement direct analysis of the aerosols, without a filter collection and reconstitution step.

We have reported increased phosphorylation rates for uridine using DAP, a prebiotically plausible phosphate source, in aerosol environments. These aerosol droplets yielded products in less than 1 h, a much faster reaction rate than previous studies that required days to observe any product formation^10^. The findings presented here have valuable implications for the study of prebiotic chemistry, as they demonstrate that such environments could offer various advantages, such as increased reaction rates and formation under mildly acidic aqueous conditions. Phosphorylation in particular has been demonstrated to overcome thermodynamic and kinetic limitations in an aqueous environment by investigating these reactions in aerosols. Studies in which aerosols are analyzed after longer incubation times may result in not only higher product yields, but oligomers may be observed as seen by Krishnamurthy and co-workers in their large-scale paste reaction^10^. Lastly, it would be of interest to investigate a DAP-based system in aerosols in which different prebiotically plausible targets are phosphorylated, such as different nucleosides, sugars, glycerol and amino acids.

## Methods and Materials

### DAP synthesis

**Figure 5:**
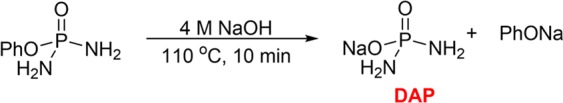
Chemical reaction scheme showing DAP synthesis.

DAP was synthesized according to a previously reported protocol as shown in Fig. 5 ^10^. A reaction mixture of phenyl phosphorodiamidate (Alfa Aesar, reagent grade 98%+) (4.9915 g, 29.0 mmol, 1.0 equiv.), NaOH (2.3998 g, 60 mmol, 2.1 equiv.) and H_2_O (15 mL) was stirred at 110 °C for 10 min. The brown solution was concentrated to 5 mL and 70 mL of ethanol was added at 0 °C. The grey precipitate was filtered and dissolved in 50 mL of cold water. The resulting solution was washed consecutively with dichloromethane twice (30 mL × 2) and ethyl acetate five times (30 mL × 5) until no phenol or phenoxide was present in the organic phase (checked by thin layer chromatography (TLC). The slightly yellowish aqueous phase was concentrated to 5 mL and was added dropwise to 50 mL of cold ethanol under vigorous stirring to afford a white precipitate. The precipitate was filtered, washed with cold ethanol for three times (20 mL × 3) and dried under vacuum overnight to afford 2.852 g (24.2 mmol, 83.4%) of DAP as a white powder. ^1^H and ^31^P NMR experiments (See Supplementary Fig. S1) were recorded on a Varian Mercury Vx 400 NMR spectrometer to determine purity. Chemical shifts (*δ*) are reported in ppm. D_2_O was used as the solvent. 85% H_3_PO_4_ (in a sealed 1 mm capillary) was used as the internal standard for ^31^P -NMR experiments.

### Solution preparation

31 mmol of DAP (corresponding to 5 eq) were dissolved in 200 mL H_2_O. To this mixture was added 1 eq uridine (Acros Organics, 99%), 3 eq MgCl_2_ (Sigma Aldrich), and the pH was adjusted to 5.5 using 4 M HCl. These conditions were based on previous work by Krishnamurthy and co-workers^10^, in which they determined that Mg^2+^ at a concentration of 3 equivalents yielded the best phosphorylation rates, and that a pH of 5.5 was optimal in the bulk and paste experiments. The effect of imidazole was investigated by adding 1 eq of imidazole (Acros Organics, 99%) to corresponding solutions before pH adjustment.

### Aerosol formation, characterization, and collection

Experiments were performed in the 1.1 m^3^ Teflon chamber at room temperature (~25 °C) under dry conditions (<5% RH). Aerosols were introduced into the chamber (Fig. 1) by atomizing the prepared solution using an atomizer in the non-recirculation mode (TSI 3076). Size-dependent particle number and volume concentrations were monitored using a Scanning Mobility Particle Sizer (SMPS). The SMPS consists of a Differential Mobility Analyzer (DMA) (TSI 3081) and a Condensation Particle Counter (CPC) (TSI 3775). Sheath flow is set to 2 L min^−1^ in order to monitor the particle size range from 18 nm to 950 nm (mobility diameter). The initial particle number concentration in the chamber was ~1 × 10^5^ particles cm^−3^. After 20 min of residence time inside the chamber, aerosol particles were collected onto 47 mm Teflon filters (Pall Laboratory) for 30 min at a flow rate of 25 L min^−1^.

### Reconstitution and LC-MS analysis

Aerosol products deposited on Teflon filters were reconstituted using 100 µL H_2_O drop-casted in the center of the filter. The filters were then incubated for 10 min at 25 °C, and the solution drop was collected for immediate LC-MS analysis.

All LC-MS analysis was performed on an Agilent 6430 Triple Quadrupole LC/MS system. Products were separated on a Waters XBridge BEH C18 2.5 µm column (2.1 × 150 mm). The following elution scheme was used for chromatographic separation: 95% 20 mM NH_4_HCO_3_ + 10 mM dibutylamine, pH 9 (A) and 5% 20 mM NH_4_HCO_3_, 10 mM dibutylamine, pH 9, 33% acetonitrile (B) for 12 min. Flow rate was 0.3 mL/min, sample injection volume was 20 µL, and column temperature was 25 °C. Absorbance was measured at 260 nm, and peak processing was performed using MassHunter Qualitative Analysis B.08.00. All peak areas were normalized. Peak retention times were found to vary slightly from run to run. This is likely due to the fact that the ion-pair reagent used must interact with not only the analyte, but with the column itself, requiring extended times to reach equilibrium with the column. Since gradient elution is necessary for these experiments, true equilibrium is not able to be reached during the course of the separations, leading to small changes in retention times between runs.

Mass spectrometry was carried out in negative ion mode with a probe capillary voltage of 4.0 kV. The spray chamber temperature was set to 300 °C, the nitrogen flow to 11 L/min, and the nebulizer gas pressure to 15 psi.

### Control experiments

Direct aerosol control experiments were performed using the typical aerosol formation setup, with the exception of no Teflon chamber. Solutions were atomized for 5 min. directly onto Teflon filters, then reconstituted in 100 µL H_2_O for LC-MS analysis.

Control experiments in which one component was deposited directly onto the Teflon filters and the other collected from the aerosol phase were also conducted. We atomized a solution of either uridine or DAP directly onto the filter for 5 min. This filter was then used as the collection surface in our typical aerosol formation setup, with a solution of either uridine or DAP atomized for 20 min into the Teflon chamber. The resulting aerosols were collected onto the Teflon filters.

Dry-down experiments were performed by drop-casting 100 µL of stock solutions without and with imidazole onto filter surfaces, incubating at 25 °C for 50 min, and reconstituting in 100 µL H_2_O.

## Supplementary information

Prebiotic Phosphorylation of Uridine using Diamidophosphate in Aerosols

## Data Availability

The datasets generated during and/or analyzed during the current study are available from the corresponding authors (T.M.O. or N.L.N.) on reasonable request.
