# Supplementary material for: Prebiotic Phosphorylation of Uridine using Diamidophosphate in Aerosols
Source: Sci Rep. 2019 Sep 19;9:13527. doi: 10.1038/s41598-019-49947-8 (PMC6753121; doi:10.1038/s41598-019-49947-8)
Supplement: Supplementary file 1 — Prebiotic Phosphorylation of Uridine using Diamidophosphate in Aerosols [file 41598_2019_49947_MOESM1_ESM.pdf]

Prebiotic Phosphorylation of Uridine using Diamidophosphate in Aerosols

A. D. Castañeda[1], Z. Li[1], T. Joo[2], K. Benham[1], B. T. Burcar[1], R. Krishnamurthy[3], C. L. Liotta[1], N. L. Ng[4,2]*, and T. M. Orlando[1]*

[1]School of Chemistry and Biochemistry, Georgia Institute of Technology, Atlanta, GA, 30332, USA

[2]School of Earth and Atmospheric Sciences, Georgia Institute of Technology, Atlanta, GA, 30332, USA

[3]Department of Chemistry, The Scripps Research Institute, La Jolla, CA, 92037, USA

[4]School of Chemical and Biomolecular Engineering, Georgia Institute of Technology, Atlanta, GA, 30332, USA

*Correspondance and requests for materials should be addressed to T.M.O. (email: thomas.orlando@chemistry.gatech.edu) or N.L.N. (email: ng@chbe.gatech.edu)

Supplementary Information

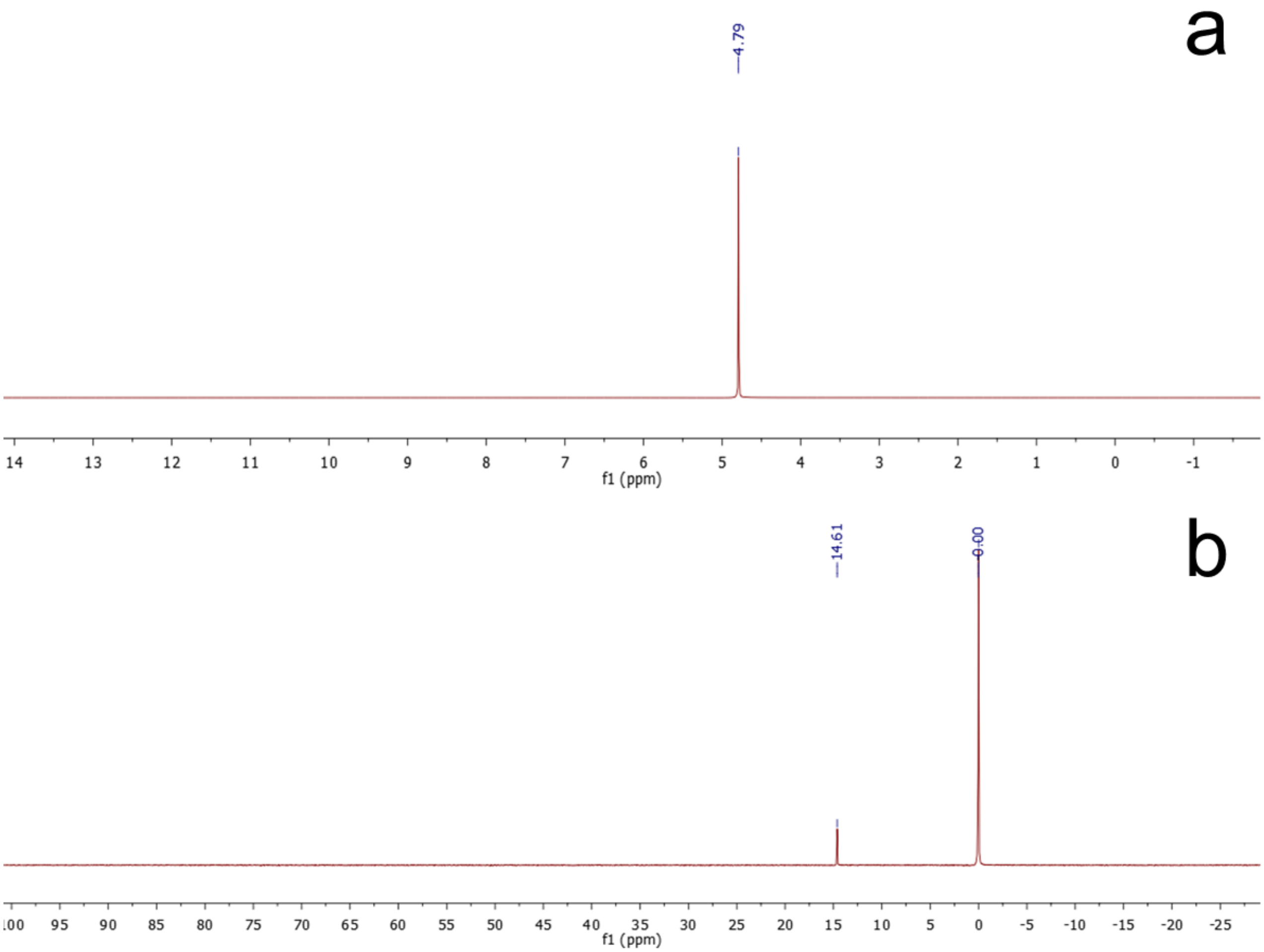

Figure S1. (a) 400 MHz ^{1}H NMR spectrum of 0.1 M DAP in D_2O. (b) 160 MHz {^{1}H-decoupled+NOE} ^{31}P NMR spectrum of 0.1 M DAP in D_2O using 85% H_3PO_4 (in a sealed 1 mm capillary) as internal standard (chemical shift = 0 ppm).

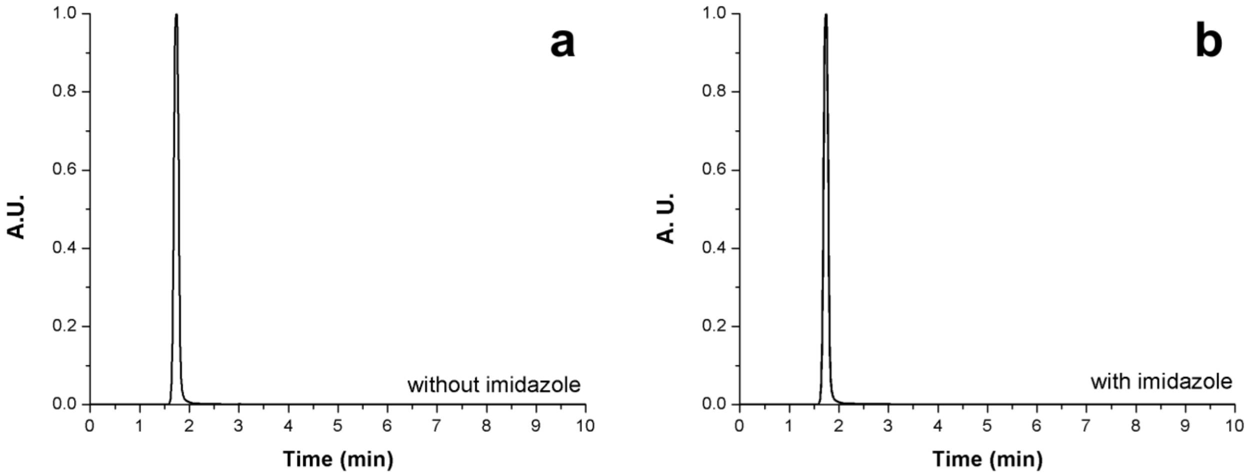

Figure S2. LC chromatograms of stock solutions consisting of 1 eq Uridine + 5 eq DAP+ 3 eq $MgCl_2$, pH adjusted to 5.5 (**a**) without and (**b**) with the addition of 1 eq imidazole. No product formation is observed.

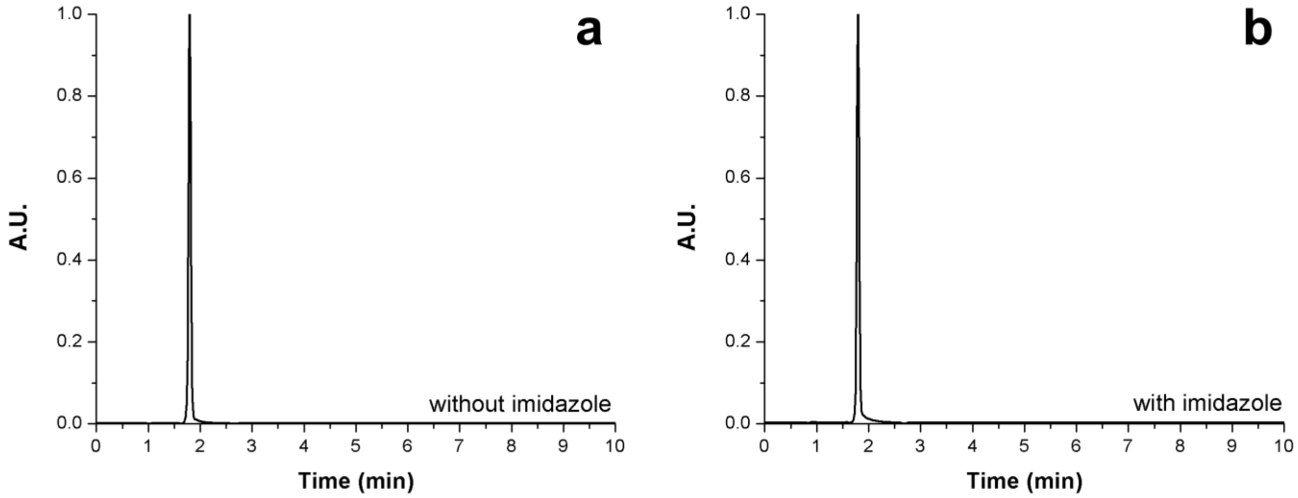

Figure S3. LC chromatograms of reconstituted stock solution (1 eq Uridine + 5 eq DAP+ 3 eq $MgCl_2$, pH adjusted to 5.5) after 100 µL is dried down at R.T. on the filter surface for 50 min. 100 µL of H2O was used to reconstitute the compounds. (**a**) without imidazole. (**b**) with the addition of 1 eq imidazole. No product was observed in either case.

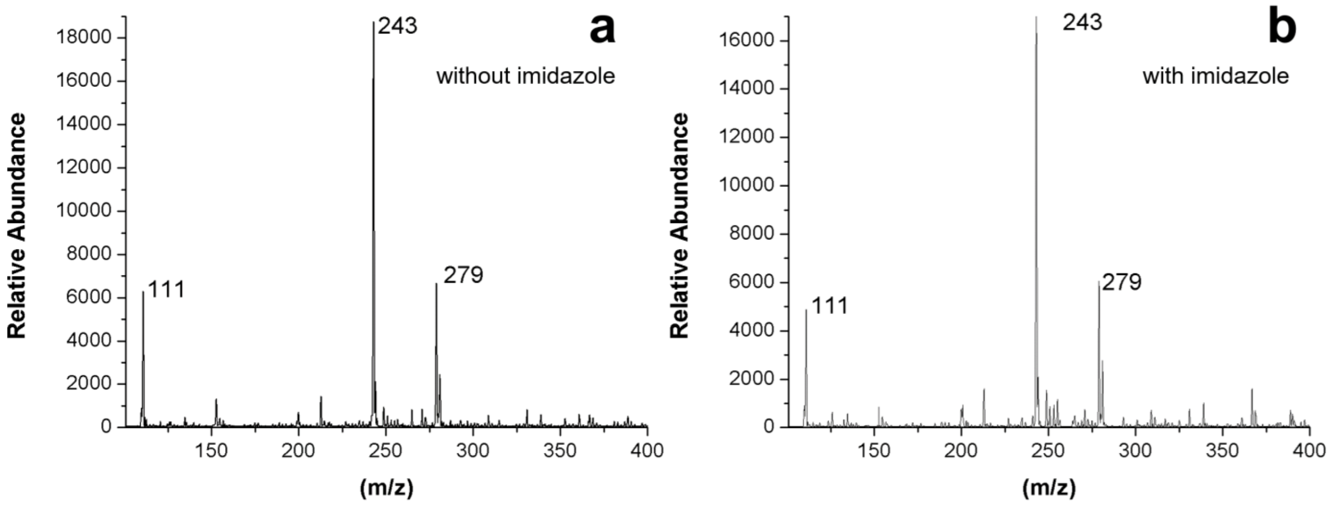

Figure S4. Direct Inject (no chromatography separation) ESI-MS of 1 eq Uridine + 5 eq DAP+ 3 eq $MgCl_2$, pH adjusted to 5.5 (**a**) without and (**b**) with addition of imidazole. No peaks corresponding to 2',3'-cUMP are observed in either case.
